# The Book World of Medicine and Science

**Published:** 1904-05-21

**Authors:** 


### THE BOOK WORLD OF MEDICINE AND SCIENCE.

BLOOD-PRESSURE IN SURGERY. By GEORGE CRILE, M.D., Professor of Clinical Surgery, Western Reserve Medical College, U.S.A., Pp. 422. Illustrations, 94. (Philadelphia and London: J. B. Lippincott Company. 1903. Price 18s. net.)

THE subject of this book is an important one in view of the strides which operative surgery has made in the last fifty years. The introduction of anæsthetics did much to reduce surgical shock, but the extensive and often prolonged operations of the present day, although performed under anæsthesia, are not infrequently associated with grave diminution in blood-pressure and its attendant evils. There is undoubtedly a considerable divergence of opinion as to the actual causation of this low blood-pressure, and perhaps even greater difference of opinion as to the proper lines of treatment of the condition. It has been the author's aim by an elaborate and carefully observed series of experiments to arrive at some authoritative conclusions in the matter. The summary of the experimental data are of the utmost value and well repay perusal. After an accurate and detailed record of no fewer than 251 experiments upon 243 animals, all thoroughly anæsthetised, Dr. Crile sums up under various headings, giving the effects of alcohol, nitroglycerine, digitalis, strychine, saline infusion, adrenalin, etc. Pressure applied externally, particularly that carried out by an ingenious pneumatic rubber suit, produced some very interesting phenomena. There are also some important pages dealing with resuscitation. A full account is given in addition of the recent methods of obtaining and recording the blood-pressure in the living human subject. The last part of the book deals with actual data observed by a study of the changes in blood-pressure during surgical operations, and charts are introduced showing these alterations in such surgical procedures as trephining, operations upon the neck, amputation of the mamma, cholecystotomy, herniotomy, amputations, etc. In his final summary the author makes a clear distinction between surgical shock and collapse. By the former he understands an exhaustion of the vaso-motor centres, there being no involvement of the heart-muscle, the cardiac centres, or the respiratory centre, except secondarily. Collapse, on the other hand, is due to a suspension of the function of the cardiac or vaso-motor mechanism, or to hæmorrhage. His conclusions as to the treatment of the two conditions are most helpful and make the work a valuable addition to our knowledge of an important subject.

THE MEDICAL ANNUAL: A YEAR BOOK OF TREATMENT AND PRACTITIONER'S INDEX. (Bristol: John Wright and Co. 1904. Pp. 839. 34 Plates. 71 Illustrations. Price 7s. 6d. Stereoscope, 2s. 6d.)

COMMENDATION of this well-known annual, now in its twenty-second year of issue, is almost superfluous. The volume before us fully maintains the high reputation of those which have preceded it, the names of the contributors being in themselves a guarantee of the thoroughness with which the sifting of the year's literature has been done. In addition to an excellent critical digest of the more important contributions to medical knowledge, the practitioner will find the annual a mine of information on such subjects as new books and appliances, sanatoria, asylums, nursing homes, and inebriate retreats, about which it is not always easy to obtain rapid information. The illustrations are excellent, of special interest being a series of plates, several in colour, illustrating the eruption in various infectious diseases, prominence being given to the distinction between small-pox and chicken-pox. An important new departure has also been made in the introduction of twelve stereoscopic pla[illegible] illustrating the anatomy of the ear in relation to the masto[illegible] operation by Dr. Kerr Love. The value of stereoscop[illegible] views for teaching purposes is now clearly recognised, a[illegible] these excellent photographs should prove of great assistan[illegible] in the study of the complicated structures involved. [illegible] medical men desirous of keeping [illegible]east of profession[illegible] knowledge, even when within reach [illegible]f a li[illegible]rary, and st[illegible] more when without that advantage, we woul[illegible] rec[illegible] the regular purchase and frequent use of this valuab[illegible] publication.

THE CITY OF LONDON DIRECTORY, 1904. (London: W.[illegible] and L. COLLINGRIDGE. Price 12s. 6d.)

THE vast quantity of information which this exceeding[illegible] useful publication contains renders it an indispensab[illegible] book for reference. The present volume, which represen[illegible] the thirth-fourth annual edition, besides a complete dir[illegible] tory for the City of London contains also much oth[illegible] interesting and instructive matter. To give some idea [illegible] the scope of the work it may be mentioned that it contai[illegible] in addition to an official and corporation directory, [illegible] commercial directory which includes the name, addre[illegible] and trade or profession of each member of firms [illegible] the City of London, a streets guide, a trades guide, [illegible] public companies directory, a conveyance director[illegible] and so forth. Not the least interesting portion [illegible] the biographical directory containing portraits and biog[illegible] phical details of the aldermen, principal city officers, a[illegible] many of the masters of the livery companies. A lar[illegible] coloured map of the City of London is also included. [illegible] note that the work has been corrected quite up to da[illegible] for the name of the late Mr. Justice Byrne has be[illegible] removed from the list of judges, and several other instanc[illegible] occur which show that revision, for practical purposes, [illegible] been made right up to the actual date of issue.

MATRICULATION DIRECTORY. No. XXXVI., January, 19[illegible] With List of Text-books for use under the New Regu[illegible] tions. (Published by W. B. CLIVE, 157 Drury La[illegible] W.C. Price 1s.)

THIS Directory is published under the auspices of t[illegible] University Correspondence College, but will be found use[illegible] to all students who propose going up for a matriculati[illegible] examination, though it is more specially arranged with [illegible] view to the University of London. The requirements for ea[illegible] degree are given, and this is followed by a list of the speci[illegible] subjects for 1905, some advice as to the best books for getti[illegible] up the subjects, and finally samples of the papers set at t[illegible] matriculation examination this year, and solutions of t[illegible] questions in these. This cheap little book can hardly f[illegible] to be useful to any young student.

